# The Soluble Guanylate Cyclase Stimulator BAY 41-2272 Attenuates Transforming Growth Factor β1-Induced Myofibroblast Differentiation of Human Corneal Keratocytes

**DOI:** 10.3390/ijms232315325

**Published:** 2022-12-05

**Authors:** Irene Rosa, Bianca Saveria Fioretto, Eloisa Romano, Matilde Buzzi, Rita Mencucci, Mirca Marini, Mirko Manetti

**Affiliations:** 1 Section of Anatomy and Histology, Department of Experimental and Clinical Medicine, University of Florence, 50134 Florence, Italy; 2 Section of Internal Medicine, Department of Experimental and Clinical Medicine, University of Florence, 50134 Florence, Italy; 3 Eye Clinic, Careggi Hospital, Department of Neurosciences, Psychology, Pharmacology and Child Health (NEUROFARBA), University of Florence, 50134 Florence, Italy; 4 Imaging Platform, Department of Experimental and Clinical Medicine, University of Florence, 50134 Florence, Italy

**Keywords:** keratocytes, human cornea, myofibroblasts, corneal fibrosis, transforming growth factor β1, soluble guanylate cyclase stimulator

## Abstract

Corneal transparency, necessary for vision and depending on the high organization of stromal extracellular matrix, is maintained by keratocytes. Severe or continuous corneal injuries determine exaggerated healing responses resulting in the formation of irreversible fibrotic scars and vision impairment. Soluble guanylate cyclase (sGC) stimulation demonstrated antifibrotic effects in both experimental fibrosis and human lung and skin fibroblasts. Here, we assessed whether sGC stimulation with BAY 41-2272 could attenuate transforming growth factor β1 (TGFβ1)-induced myofibroblast differentiation of human corneal keratocytes. Cells were challenged with TGFβ1, with/without BAY 41-2272 preincubation, and subsequently assessed for viability, proliferation, migration, chemoinvasion, as well for the expression of myofibroblast/fibroblast activation markers and contractile abilities. Treatment with BAY 41-2272 did not affect keratocyte viability, while preincubation of cells with the sGC stimulator was able to inhibit TGFβ1-induced proliferation, wound healing capacity, and invasiveness. BAY 41-2272 was also able to attenuate TGFβ1-induced myofibroblast-like profibrotic phenotype of keratocytes, as demonstrated by the significant decrease in *ACTA2*, *COL1A1*, *COL1A2*, *FN1* and *PDPN* gene expression, as well as in α-smooth muscle actin, α-1 chain of type I collagen, podoplanin, vimentin and N-cadherin protein expression. Finally, BAY 41-2272 significantly counteracted the TGFβ1-induced myofibroblast-like ability of keratocytes to contract collagen gels, reduced phosphorylated Smad3 protein levels, and attenuated gene expression of proinflammatory cytokines. Collectively, our data show for the first time that BAY 41-2272 is effective in counteracting keratocyte-to-myofibroblast transition, thus providing the rationale for the development of sGC stimulators as novel promising modulators of corneal scarring and fibrosis.

## 1. Introduction

The cornea is a dome-shaped transparent tissue constituting the front ocular surface composed of three cellular layers: an external epithelium, a central avascular stromal layer accounting for more than 90% of the corneal thickness and composed of a collagen-rich extracellular matrix (ECM) intermingled with interstitial cells, and an internal layer of endothelial cells. The Bowman’s membrane lays between the epithelial and the stromal layer, while the Descemet’s membrane separates the posterior part of the stroma and the endothelial layer [1,2]. Considering its pivotal role in contributing to nearly two-thirds of the eye’s total focusing power, cornea has to be perfectly clear and preserve a smooth and stable curvature. Corneal transparency is given by the high organization of stromal ECM, which is indeed composed of bundles of collagen fibers closely packed in lamellae parallel arranged to the corneal surface [1,2]. Of note, the synthesis/deposition of collagen fibrils during development and the ongoing maintenance of the uniform alignment of lamellae are regulated by keratocytes, neural crest-derived fibroblastic cells representing the main cell type within the stromal layer [1,2].

Injuries to the cornea induce complex wound healing responses to protect and restore the normal corneal structure and transparency [2,3,4,5]. In particular, after corneal damage, stromal-resident keratocytes, which are normally in a quiescent state, are “triggered” by epithelial-derived cytokines, chemokines, and growth factors such as transforming growth factor β (TGFβ) and platelet-derived growth factor (PDGF), and transform into corneal fibroblasts, activated cells that secrete relatively small amounts of disordered ECM and are able to revert to the keratocyte phenotype as the wound healing response subsides in the stroma [2,6,7]. In case of sustained exposure to TGFβ, corneal keratocyte-derived fibroblasts are instead further triggered to differentiate into myofibroblasts, α-smooth muscle actin (α-SMA)-expressing cells that produce large amounts of ECM [2,6,8]. In the event of minor abrasions, myofibroblasts disappear by apoptosis after restoring tissue integrity, and corneal transparency is maintained. Severe or continuous insults can instead determine myofibroblast persistence, thus leading to the deposition of excessive and irregular ECM components, and finally resulting in the formation of an irreversible fibrotic scar accounting for corneal opacity and consequent vision impairment. Such scars can be caused by improper use of contact lenses, deep scratches, lacerations, burns, and some diseases such as shingles and syphilis [2,6]. Other factors that have been associated with corneal fibrosis include genetic variations, dietary problems, and environmental influences such as ultraviolet light exposure [9]. Even if various therapeutic options for corneal stromal regeneration (e.g., stem cell therapy, biomaterials, growth factors, tissue engineering, and replacement of diseased or damaged corneal tissue) have been developed to improve damaged human vision, the use of such treatments is challenging and has encountered numerous hurdles including significant potentially blinding side effects [2,10,11,12]. In this context, the modulation of myofibroblast differentiation in an injured cornea represents a critical therapeutic target to minimize corneal scarring and preserve clear vision.

Soluble guanylate cyclase (sGC) is an enzyme that catalyzes cyclic guanosine monophosphate (cGMP) production following the binding of nitric oxide (NO) to its prosthetic heme group, thus regulating a variety of downstream processes including cell growth and proliferation, immune responses, and ECM synthesis [13]. Increasing sGC activity and consequent cGMP production with selective stimulators such as BAY 41-2272, BAY 60-277, riociguat, and MK-2947 was reported to exert antifibrotic effects in vivo and/or in vitro [14,15,16,17,18,19,20,21,22,23,24]. In particular, treatment with BAY 60-277 dampened hepatic fibrosis in different rat models [14,15], while BAY 41-2272 decreased experimental cardiac and dermal fibrosis [17,20,22]. Another sGC stimulator, named riociguat, was also effective in reducing collagen deposition and myofibroblast accumulation in experimental skin fibrosis of different etiologies [22], as well as to ameliorate gastrointestinal fibrosis in the bleomycin-induced mouse model [23]. In vitro, BAY 41-2272 significantly attenuated the TGFβ1-induced gene and protein expression of the fibrogenic cytokine connective tissue growth factor in mouse primary hepatic stellate cells [16], and lessened TGFβ-induced cardiac, lung, and dermal fibroblast-to-myofibroblast differentiation [17,18,19,20,21]. In addition, sGC stimulation with MK-2947 blunted the myofibroblast-like features of systemic sclerosis endothelial cells, which are known to undergo an endothelial-to-mesenchymal transition [24]. Nevertheless, the role of sGC stimulation in corneal fibrosis, and especially in myofibroblast differentiation from quiescent keratocytes, had yet to be investigated. On these bases, the present study was performed to assess if the stimulation of sGC with BAY 41-2272 was able to attenuate TGFβ1-induced myofibroblast differentiation of human corneal keratocytes.

## 2. Results

### 2.1. Isolated Human Corneal Keratocyte Morphology Assessment

**Figure 1:**
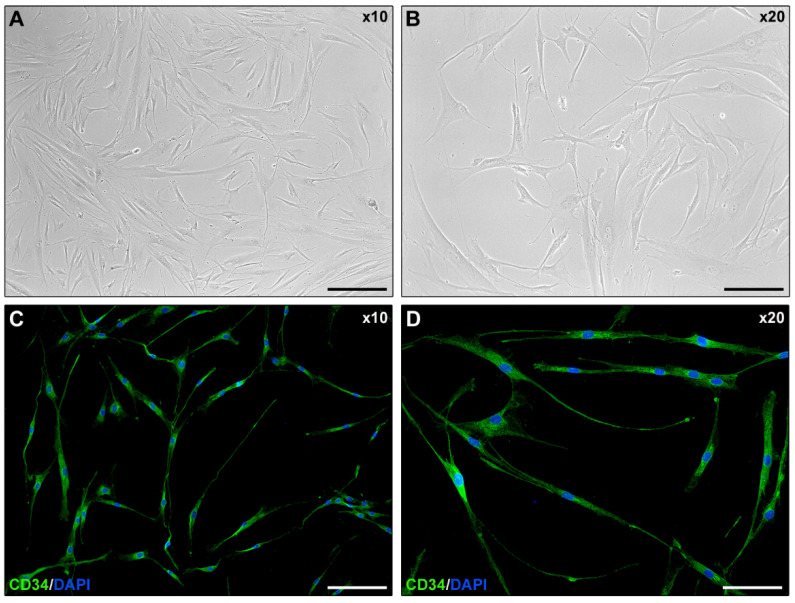
Morphological and immunophenotypical features of explanted and in vitro cultured normal human corneal keratocytes. Representative phase-contrast (**A**,**B**) and CD34 immunofluorescence (**C**,**D**) photomicrographs of keratocytes at first passage in culture. Original magnification: ×10 (**A**,**C**), ×20 (**B**,**D**). Scale bar: 200 μm (**A**,**C**), 100 μm (**B**,**D**).

Normal human keratocytes were isolated from eye bank cadaveric donor corneal tissues (*n* = 5) unsuitable for clinical use. As shown in Figure 1A,B, explanted corneal keratocytes displayed a stellate morphology with long cytoplasmic processes extending from the cell body. Moreover, freshly isolated keratocytes displayed immunopositivity for the keratocyte marker CD34 [2].

### 2.2. BAY 41-2272 Does Not Affect Viability and Inhibits TGFβ1-Induced Proliferation of Human Corneal Keratocytes

It is well established that TGFβ plays a pivotal role in the conversion of keratocytes into myofibroblasts, fostering α-SMA expression, cell proliferation, and migration [4,25,26,27]. For this reason, to assess whether sGC stimulation could attenuate TGFβ-induced profibrotic and contractile phenotype in corneal keratocytes, cells were challenged with BAY 41-2272 (10 µM) alone or in combination with recombinant human TGFβ1 (10 ng/mL) in all sets of experiments.

**Figure 2:**
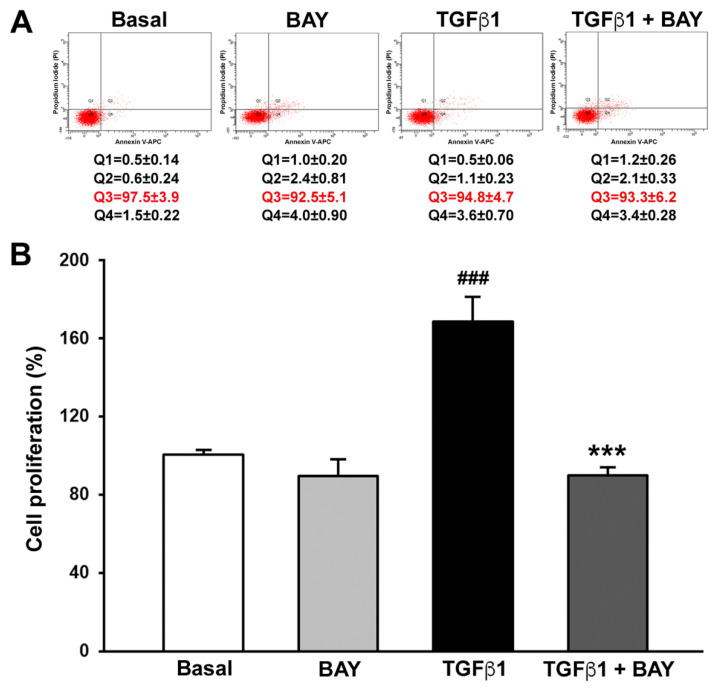
Soluble guanylate cyclase stimulation with BAY 41-2272 does not affect human keratocyte viability and significantly inhibits TGFβ1-induced cell proliferation. (**A**) Representative annexin V/PI flow cytometry assay plots of keratocytes at basal condition and after treatment with recombinant human TGFβ1, TGFβ1 and BAY 41-2272, or BAY 41-2272 alone. Viable cells (annexin V^−^/PI^−^), early apoptotic cells (annexin V^+^/PI^−^), late apoptotic cells (annexin V^+^/PI^+^), and necrotic cells (annexin V^−^/PI^+^) are represented in the lower left (Q3), lower right (Q4), upper right (Q2), and upper left (Q1) quadrant, respectively. The mean ± SEM percentage of necrotic (Q1), late apoptotic (Q2), viable (Q3, highlighted in red), and early apoptotic (Q4) cells is reported for each experimental point. (**B**) Cell proliferation determined by WST-1 colorimetric assay. Proliferation of keratocytes at basal condition was set to 100%, and the other results are normalized to this value. Bars represent the mean ± SEM of triplicate determinations from five cell lines. ### *p* < 0.001 vs. basal condition, *** *p* < 0.001 vs. TGFβ1 alone (Tukey’s test). PI, propidium iodide; SEM, standard error of the mean; TGFβ1, transforming growth factor β1.

Firstly, to verify if treatment with the sGC stimulator BAY 41-2272 could have any possible side effects, cell viability and proliferation were determined by annexin V/propidium iodide (PI) flow cytometry and WST-1, respectively. Annexin V/PI assay is indeed used to discriminate viable, early apoptotic, late apoptotic, or necrotic cells on the bases of differences in their plasma membrane integrity and permeability, while WST-1 assay allows the evaluation of cell proliferation by measuring cell metabolism. As shown in Figure 2A, treatment with TGFβ1 or BAY 41-2272, both alone and in combination, did not affect cell viability. In fact, no significant differences in the percentage of viable, early apoptotic, late apoptotic, or necrotic cells were detected among the different experimental points (Figure 2A). As far as cell proliferation is concerned, BAY 41-2272 alone had no effect, while stimulation with TGFβ1 alone determined a significant increase in keratocyte proliferation. Notably, keratocytes preincubated with BAY 41-2272 and then treated with TGFβ1 showed a proliferation rate normalized to basal levels (Figure 2B).

### 2.3. BAY 41-2272 Reduces TGFβ1-Induced Wound Healing Capacity of Human Corneal Keratocytes

**Figure 3:**
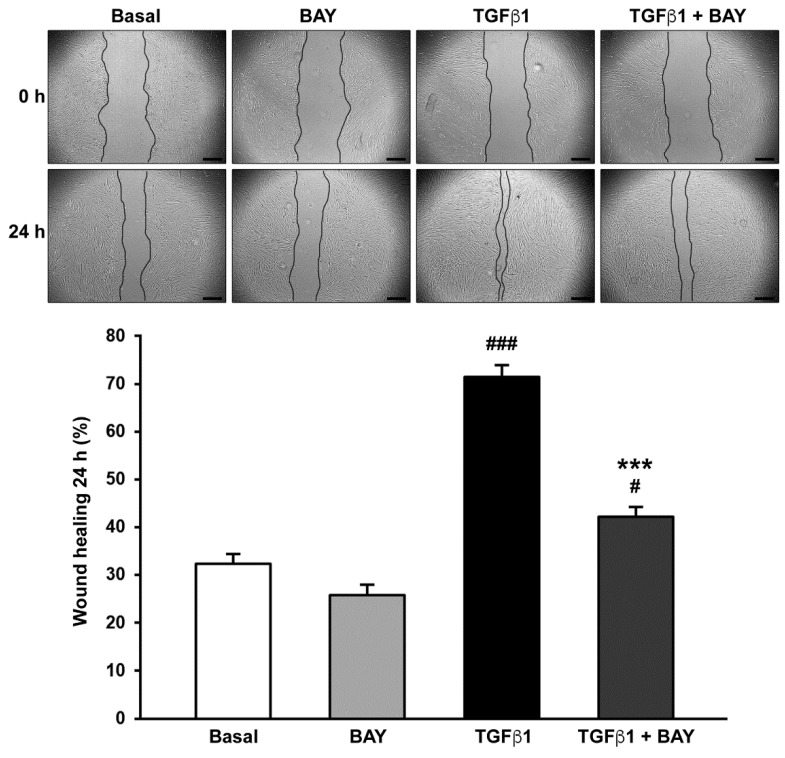
Soluble guanylate cyclase stimulation with BAY 41-2272 significantly reduces TGFβ1-induced ability of human keratocytes to restore the monolayer integrity after scratching. Wound healing capacity was evaluated in keratocytes at basal condition and after treatment with recombinant human TGFβ1, TGFβ1 and BAY 41-2272, or BAY 41-2272 alone. Representative phase-contrast photomicrographs of the wounded area at 0 and 24 h are shown for each experimental condition (original magnification ×10); wound margins are outlined in black. Scale bar: 400 μm. Bars represent the mean ± SEM of triplicate determinations of the percentage of wound healing after 24 h (*n* = 5 cell lines). # *p* < 0.05, ### *p* < 0.001 vs. basal condition, *** *p* < 0.001 vs. TGFβ1 alone (Tukey’s test). SEM, standard error of the mean; TGFβ1, transforming growth factor β1.

Keratocyte migration and proliferation abilities were simultaneously assessed with the wound healing assay performed without any mitosis inhibitor. A cell-free area was created in the confluent monolayer by mechanically removing cells with a sterile pipette tip, and the wound healing percentage was recorded 24 h after scratching. As displayed in Figure 3, in the presence of BAY 41-2272 alone, the ability of keratocytes to restore the monolayer integrity was similar to the basal condition. Conversely, stimulation with TGFβ1 alone caused a significant increase in keratocyte wound healing capacity, resulting into ~75% wound closure (*p* < 0.001 vs. basal; Figure 3). TGFβ1 stimulatory effect was significantly attenuated when cells were preincubated with BAY 41-2272 (*p* < 0.001 vs. TGFβ1 alone; Figure 3).

### 2.4. Stimulation with BAY 41-2272 Reduces TGFβ1-Induced Invasiveness of Human Corneal Keratocytes

**Figure 4:**
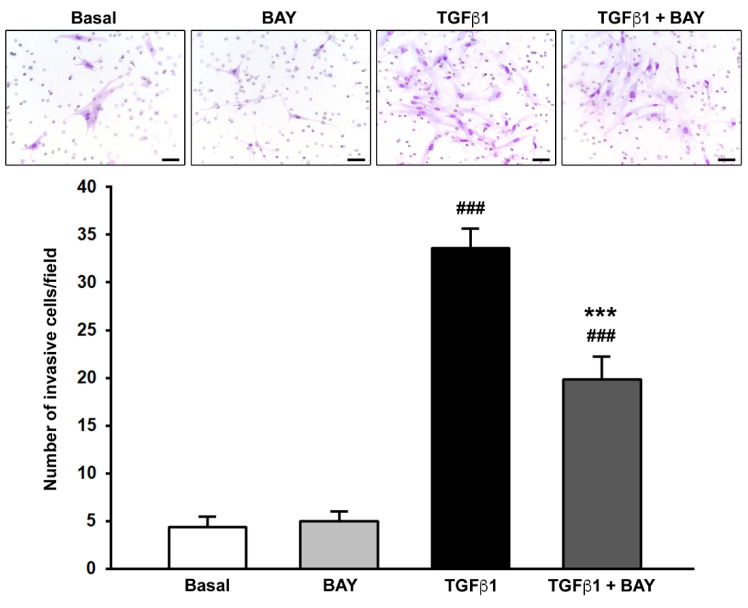
Soluble guanylate cyclase stimulation with BAY 41-2272 significantly reduces TGFβ1-induced invasiveness of human keratocytes in the Matrigel chemoinvasion assay. Representative images of the filters with invasive cells stained with Diff-Quik are shown (original magnification: ×40). Scale bar: 50 μm. Histograms represent the results of quantitative analysis of chemoinvasion expressed as the number of migrated cells per field. Data are mean ± SEM of triplicate determinations from five cell lines. ### *p* < 0.001 vs. basal condition, *** *p* < 0.001 vs. TGFβ1 alone (Tukey’s test). SEM, standard error of the mean; TGFβ1, transforming growth factor β1.

Since during in vivo corneal wound healing keratocytes migrate within the surrounding matrix [2,8], the Boyden chamber chemoinvasion assay was next performed to evaluate keratocyte capability to invade a polymeric extracellular matrix (i.e., Matrigel). As shown in Figure 4, when compared to the basal condition, the invasiveness of keratocytes was not influenced by treatment with BAY 41-2272 alone, while it was significantly augmented in the presence of TGFβ1 (*p* < 0.001). Preincubation of TGFβ1-treated keratocytes with BAY 41-2272 strongly decreased the number of invasive cells (*p* < 0.001 vs. TGFβ1 alone).

### 2.5. Stimulation with BAY 41-2272 Attenuates TGFβ1-Induced Myofibroblast-like Profibrotic and Contractile Phenotype of Human Corneal Keratocytes

**Figure 5:**
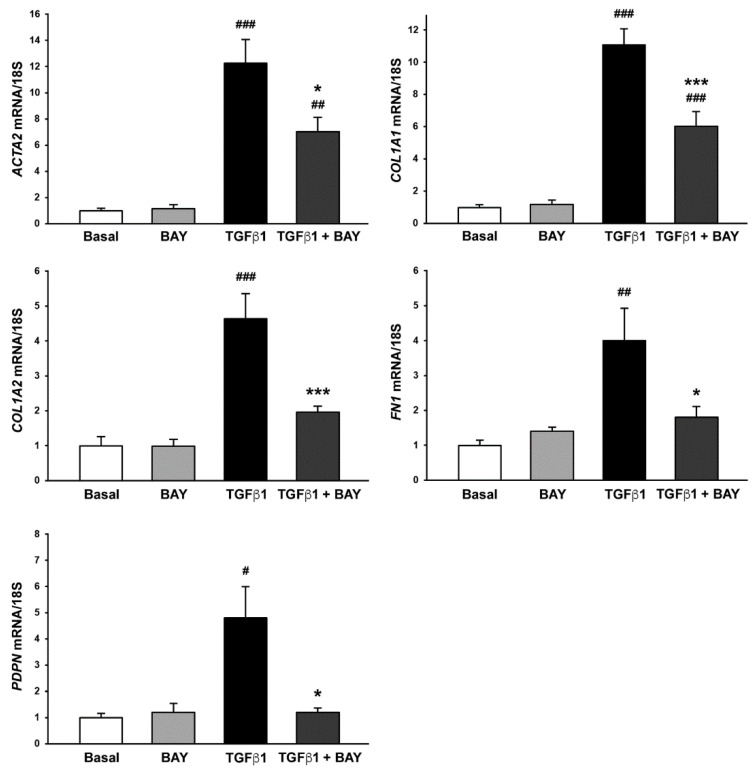
The soluble guanylate cyclase stimulator BAY 41-2272 significantly reduces TGFβ1-induced expression of genes encoding activated fibroblast/myofibroblast markers in human keratocytes. Gene expression of *ACTA2* (gene encoding α-smooth muscle actin), *COL1A1* (gene encoding α-1 chain of type I collagen), *COL1A2* (gene encoding α-2 chain of type I collagen), *FN1* (gene encoding fibronectin), and *PDPN* (gene encoding podoplanin) was evaluated by real-time PCR. The basal level of each gene expression was set to 1, and the other results are normalized to this value. 18S ribosomal RNA was used as reference gene. Bars represent the mean ± SEM of triplicate determinations from five cell lines. # *p* < 0.05, ## *p* < 0.01, ### *p* < 0.001 vs. basal condition, * *p* < 0.05, *** *p* < 0.001 vs. TGFβ1 alone (Tukey’s test). SEM, standard error of the mean; TGFβ1, transforming growth factor β1.

To evaluate whether sGC stimulation could modify TGFβ1-induced profibrotic phenotype in human corneal keratocytes, cells challenged with BAY 41-2272 were assayed for the expression of activated fibroblast/myofibroblast markers and for the ability to contract collagen gels. As expected, quantitative real-time PCR analysis performed on keratocytes stimulated with TGFβ1 alone revealed a significant increase in the expression of *ACTA2* (gene encoding α-SMA), *COL1A1* (gene encoding α-1 chain of type I collagen), *COL1A2* (gene encoding α-2 chain of type I collagen), *FN1* (gene encoding fibronectin 1), and *PDPN* (gene encoding podoplanin) genes (Figure 5). Preincubation of cells with BAY 41-2272 was able to significantly reduce TGFβ1-induced mRNA expression levels of all the aforementioned genes (Figure 5). In particular, keratocytes preincubated with BAY 41-2272 before treatment with TGFβ1 showed gene expression levels of *COL1A2*, *FN1*, and *PDPN* comparable to those of basal cells (Figure 5).

**Figure 6:**
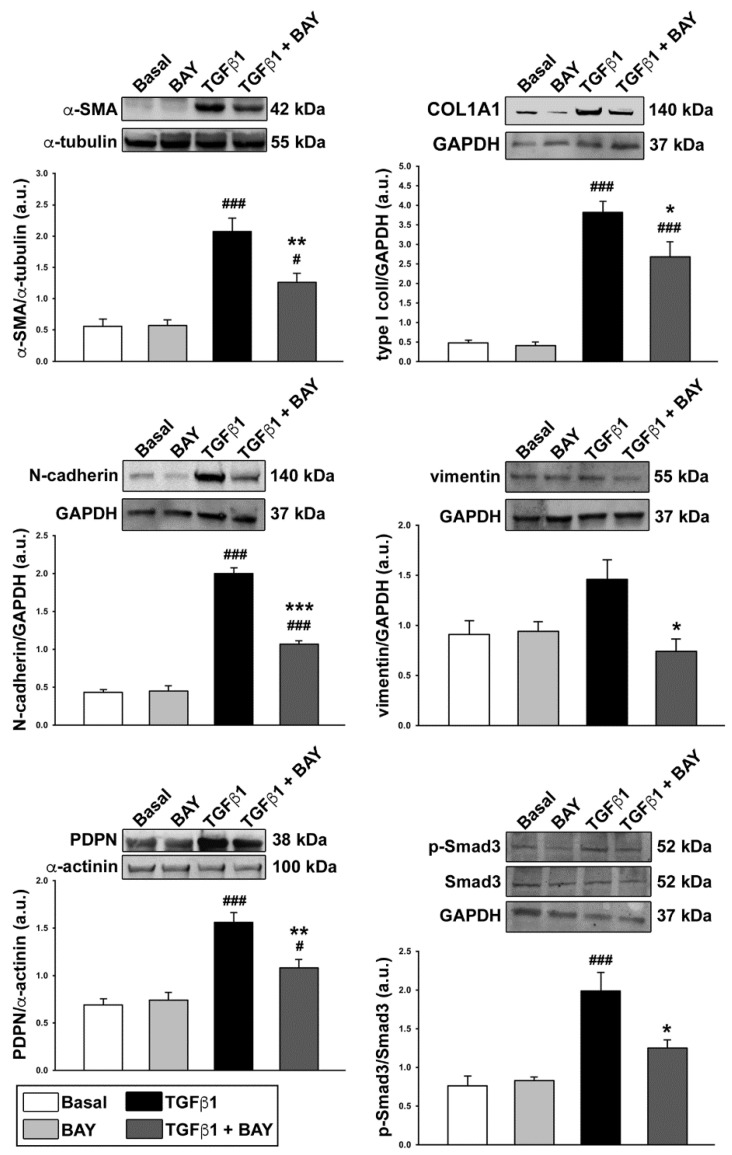
The soluble guanylate cyclase stimulator BAY 41-2272 significantly reduces TGFβ1-induced protein expression of activated fibroblast/myofibroblast markers and canonical Smad3-dependent TGFβ1 signaling in human keratocytes. Representative immunoblots for α-SMA, COL1A1, N-cadherin, vimentin, PDPN, p-Smad3, and total Smad3 are shown. α-tubulin, GAPDH or α-actinin were measured as loading controls for normalization. Molecular weight values (kDa) are indicated. Bars represent the mean ± SEM of optical density in arbitrary units (a.u.). The assay was performed in duplicate for each of the five cell lines. # *p* < 0.05, ### *p* < 0.001 vs. basal condition, * *p* < 0.05, ** *p* < 0.01, *** *p* < 0.001 vs. TGFβ1 alone (Tukey’s test). α-SMA, α-smooth muscle actin; COL1A1, α-1 chain of type I collagen; GAPDH, glyceraldehyde 3-phosphate dehydrogenase; PDPN, podoplanin; p-Smad3, phosphorylated Smad3; SEM, standard error of the mean; TGFβ1, transforming growth factor β1.

Accordingly, Western blot analysis revealed that α-SMA, α-1 chain of type I collagen (COL1A1) and podoplanin protein expression levels were augmented in TGFβ1-treated keratocytes (Figure 6). A similar significant increase was shown for vimentin and N-cadherin, two myofibroblast markers that have been previously reported to be strongly increased during myofibroblast differentiation after corneal injury or TGFβ stimulation (Figure 6) [28,29]. The TGFβ1-induced upregulation of these activated fibroblast/myofibroblast markers was significantly attenuated in cells preincubated with BAY 41-2272 (Figure 6). Since Smad3 phosphorylation represents an important step of the TGFβ1 canonical pathway, we also assessed the expression level of phosphorylated Smad3 (p-Smad3)/Smad3 in our different experimental conditions. As expected, exposure of keratocytes to TGFβ1 alone strongly increased Smad3 phosphorylation, while preincubation with BAY 41-2272 significantly reduced p-Smad3 (*p* < 0.05 vs. TGFβ1 alone), reaching levels not significantly different from those of the basal condition (Figure 6).

**Figure 7:**
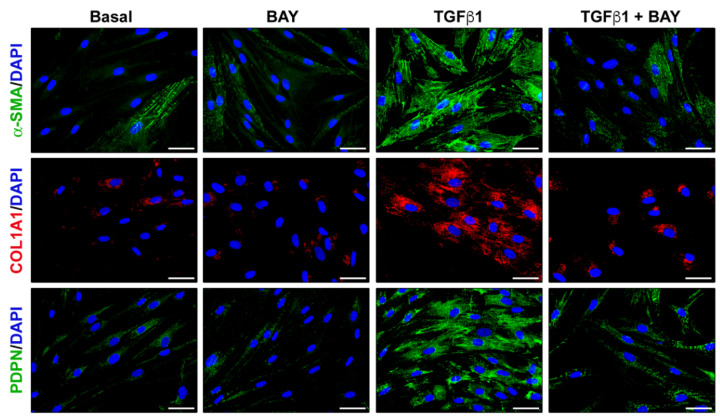
The soluble guanylate cyclase stimulator BAY 41-2272 significantly reduces TGFβ1-induced α-SMA stress fibers, COL1A1 synthesis, and PDPN immunostaining in human keratocytes. Representative fluorescence photomicrographs of keratocytes immunostained for α-SMA (green), COL1A1 (red), and PDPN (green) are shown. Nuclei are counterstained with 4′,6-diamidino-2-phenylindole (DAPI; blue). The assay was performed in duplicate for each of the five cell lines. Scale bar = 50 μm. α-SMA, α-smooth muscle actin; COL1A1, α-1 chain of type I collagen; PDPN, podoplanin; TGFβ1, transforming growth factor β1.

The attenuating effect of BAY 41-2272 on TGFβ1-induced α-SMA, COL1A1, and podoplanin protein expression was further confirmed by immunofluorescence staining (Figure 7).

**Figure 8:**
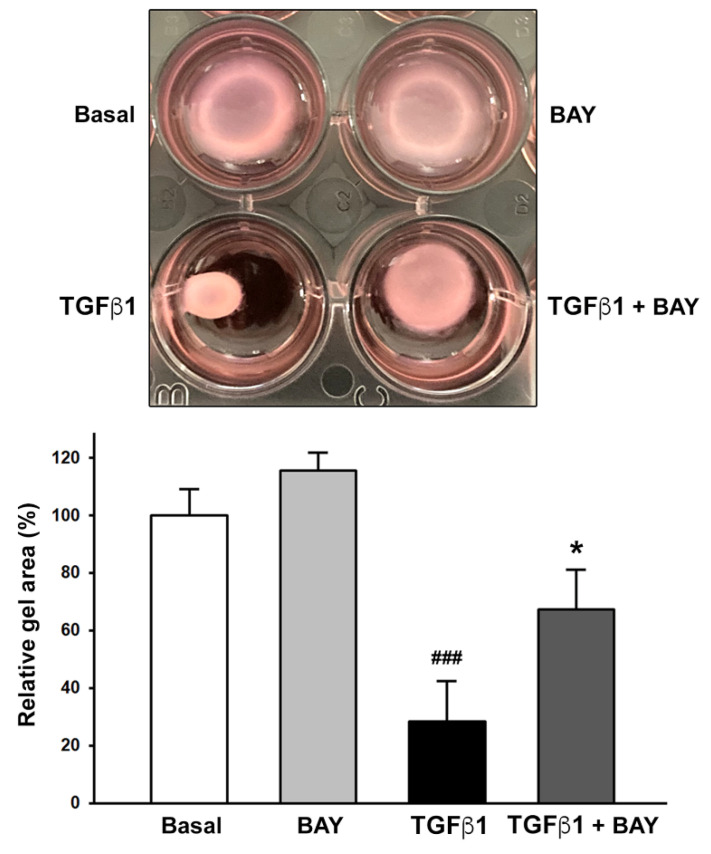
Soluble guanylate cyclase stimulation with BAY 41-2272 significantly reduces TGFβ1-induced contractile ability of human keratocytes evaluated by collagen gel contraction assay. Each experimental point was performed in triplicate for the five cell lines. Bars represent the mean ± SEM of gel sizes expressed as a percentage of that observed with cells at the basal condition; ### *p* < 0.001 vs. basal condition, * *p* < 0.05 vs. TGFβ1 alone (Tukey’s test). SEM, standard error of the mean; TGFβ1, transforming growth factor β1.

Preincubation of cells with BAY 41-2272 was also able to significantly counteract the TGFβ1-induced myofibroblast-like ability of keratocytes to contract collagen gels (*p* < 0.05 vs. TGFβ1 alone, Figure 8). Of note, the collagen gel area was similar for cells preincubated with BAY 41-2272 before treatment with TGFβ1 and basal cells (Figure 8).

### 2.6. Stimulation with BAY 41-2272 Reduces TGFβ1-Induced Expression of Genes Encoding Proinflammatory Cytokines in Human Keratocytes

**Figure 9:**
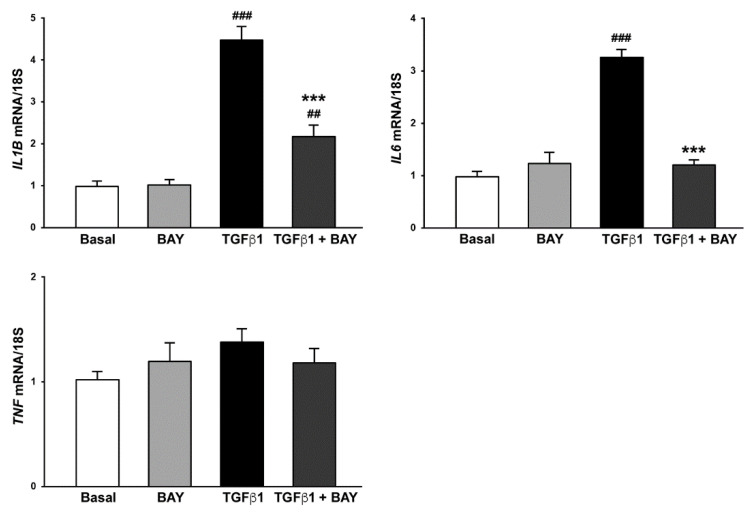
The soluble guanylate cyclase stimulator BAY 41-2272 significantly reduces TGFβ1-induced expression of genes encoding proinflammatory cytokines in human keratocytes. Gene expression of *IL1B* (gene encoding interleukin 1β), *IL6* (gene encoding interleukin 6), and *TNF* (gene encoding tumor necrosis factor α) was evaluated by real-time PCR. The basal level of each gene expression was set to 1, and the other results are normalized to this value; 18S ribosomal RNA was used as reference gene. Bars represent the mean ± SEM of triplicate determinations from five cell lines. ## *p* < 0.01, ### *p* < 0.001 vs. basal condition, *** *p* < 0.001 vs. TGFβ1 alone (Tukey’s test). SEM, standard error of the mean; TGFβ1, transforming growth factor β1.

It is well known that after corneal injury, activated keratocytes at the injured site release proinflammatory factors in order to recruit immune cells and regulate the healing process [5,27,30,31,32]. Although inflammation facilitates cell migration in order to accelerate wound healing, the overexpression of inflammatory cytokines results in corneal scarring [5,27,30,31,32]. On these bases, we finally investigated the effects of BAY 41-2272 on TGFβ1-induced proinflammatory phenotype of human corneal keratocytes. As shown in Figure 9, stimulation with TGFβ1 resulted in a strong production of *IL1B* (gene encoding interleukin 1β) and *IL6* (gene encoding interleukin 6) mRNA, an increase that was significantly attenuated in cells prestimulated with BAY 41-2272 (both *p* < 0.001 vs. TGFβ1 alone). In particular, keratocytes preincubated with BAY 41-2272 before treatment with TGFβ1 displayed *IL6* mRNA levels similar to those of basal cells (Figure 9). Conversely, no effect on *TNF* gene expression was detected (Figure 9).

## 3. Discussion

This in vitro study is the first to investigate the possible effects of pharmacological stimulation of sGC in modulating TGFβ1-triggered differentiation of human corneal keratocytes into myofibroblasts. Our results clearly demonstrated the capability of BAY 41-2272 to hamper proliferation, migration, invasiveness and profibrotic phenotype of human corneal keratocytes challenged with TGFβ1, at least partly by inhibiting canonical Smad3-dependent signaling. Moreover, BAY 41-2272 was also effective in attenuating TGFβ1-induced expression of genes encoding proinflammatory cytokines such as interleukin 1β and interleukin 6.

Myofibroblast differentiation of human keratocytes plays a pivotal role in the corneal wound healing process. In particular, the development/persistence of myofibroblasts from these stromal precursor cells depends on an adequate ongoing supply of growth factors such as TGFβ and PDGF [33]. In normal cornea, the low levels of TGFβ1 produced by epithelial cells are not able to penetrate into the stroma and to drive myofibroblast generation due to the presence of an intact epithelial basement membrane (EBM). In case of minor injuries such as corneal abrasions, TGFβ1 leaks through the damaged EBM and penetrates into the stroma, where it triggers the development of myofibroblasts from quiescent keratocytes [6,33,34]. Nevertheless, since the EBM integrity is quickly regenerated, myofibroblasts undergo apoptosis before producing sufficient disordered ECM to reduce corneal transparency [6,35]. Upon a more severe injury, the EBM regeneration may be conversely delayed, allowing ongoing penetration of TGFβ1 from the epithelium into the stroma [6,35]. Therefore, myofibroblasts escape apoptosis and persist in the stroma, leading to the deposition of excessive and irregular ECM components, and finally resulting in the formation of an irreversible fibrotic scar [4,5,6].

Growing literature demonstrated that an increase in cGMP levels determined by sGC stimulation/activation can prevent TGFβ-induced myofibroblast differentiation in different stromal cells including human dermal and lung fibroblasts [18,19,20,21]. Moreover, sGC stimulation was shown to inhibit both epithelial- and endothelial-to-mesenchymal transition [24,36]. Recently, an in vitro study performed on cultured human keratocytes exposed to TGFβ showed that the stimulation of these activated cells with exogenous NO was able to decrease myofibroblast differentiation [29]. Interestingly, the addition of an sGC inhibitor eliminated the anti-myofibroblastic effect of NO, suggesting that cGMP production through sGC had a critical role in NO-mediated prevention of myofibroblast differentiation from keratocytes [29]. Based on these promising results, herein we aimed to evaluate the effects of a direct pharmacological stimulation of sGC on TGFβ1-triggered differentiation of human corneal keratocytes into myofibroblasts. Considering that an ideal therapeutic agent able to prevent corneal haze should be safe for keratocytes, we first investigated whether pharmacological sGC stimulation could have a potentially damaging effect on cells, and we demonstrated that BAY 41-2272 did not affect keratocyte viability. Such a result agrees with the aforementioned study of Park et al., where no toxic outcome was detected for exogenous NO as well [29]. Interestingly, we also reported for the first time that sGC stimulation can significantly inhibit TGFβ1-induced keratocyte proliferation, an effect that has been previously demonstrated in human lung fibroblasts [18]. Besides increasing cell proliferation rate, stimulation of keratocytes with TGFβ is also well established to lead to the acquisition of myofibroblast hallmarks by enhancing their migratory and invasive behavior, as well as by fostering α-SMA-dependent contractile forces [27,29,37]. In our study, preincubation of TGFβ1-treated keratocytes with BAY 41-2272 was able to significantly counteract exaggerated wound healing ability and invasiveness, as well as to reduce the expression of activated fibroblast/myofibroblast markers such as α-SMA, thus finally resulting in a reduced contractile phenotype. This is in line with a previous study showing that activation of the NO/cGMP signaling pathway significantly decreased the expression of α-SMA and N-cadherin mesenchymal markers in TGFβ1-stimulated keratocytes [29]. TGFβ-induced expression of αSMA and type I collagen was also reported to be dampened by pharmacological sGC stimulation/activation of lung, prostatic and dermal fibroblasts [18,19,20,21]. Interestingly, in a previous study from our group, sGC stimulation was able to blunt the contractile features of systemic sclerosis endothelial cells, which are known to exhibit a myofibroblast-like profibrotic phenotype [24]. Although previous evidence indicated that pharmacological sGC stimulation is effective in inhibiting non-canonical TGFβ signaling in both human dermal fibroblasts and experimental skin fibrosis [21,22], on the bases of two recently published works reporting that Smad3-dependent canonical pathway was triggered in corneal fibroblasts challenged with TGFβ1 [27,38], we evaluated the protein expression of p-Smad3 in our different experimental conditions. Interestingly, we demonstrated for the first time, that in keratocytes, BAY 41-2272 counteracted TGFβ1-induced cell profibrotic features through the reduction of p-Smad3 protein levels. However, we cannot exclude that sGC stimulation could interfere also with the non-canonical, Smad-independent TGFβ signaling pathways in keratocytes [7]. Moreover, we found that BAY 41-2272 was effective in dampening TGFβ1-triggered upregulation of podoplanin, a marker of fibroblast activation and invasiveness that, to the best of our knowledge, has never been described before in corneal keratocytes [39,40]. Noteworthy, our findings also showed that besides exerting antifibrotic effects, BAY 41-2272 was able to attenuate TGFβ1-induced proinflammatory phenotype of human corneal keratocytes by reducing *IL1B* and *IL6* gene expression. This is in line with other studies reporting anti-inflammatory properties of sGC stimulation in different experimental models of fibrosis and inflammation [41,42,43].

Collectively, our data show that treatment with BAY 41-2272 is effective in counteracting corneal keratocyte-to-myofibroblast transition, thus providing the rationale for the development of sGC stimulators, in the form of eye drops for topical application, as novel promising modulators of corneal scarring and fibrosis in the treatment of various ocular diseases threatening corneal transparency. Noteworthy, sGC stimulators have already shown beneficial effects with good tolerability in the treatment of different types of experimental fibrosis and are already in clinical practice for the treatment of some human conditions [14,15,17,20,22,23,24]. However, since our current research is based on in vitro experiments, which are not able to fully reproduce the complexity of the human corneal microenvironment, we are aware that further preclinical studies will be necessary to confirm if our findings will be repeatable also in vivo, such as in the mouse model of corneal opacity development after alkali burn [29]. In such a perspective, we are confident that these promising results will provide the necessary groundwork for further investigations on the feasibility of sGC stimulation as novel therapeutic approach to fight corneal fibrosis.

## 4. Materials and Methods

### 4.1. Isolation of Normal Human Corneal Keratocytes

Primary cultures of normal human corneal keratocytes were established from eye bank cadaveric donor corneal tissues (*n* = 5) unsuitable for clinical use provided by Fondazione Banca degli Occhi del Veneto Onlus, Venice, Italy. According to eye bank regulations, cadaveric donor corneal tissues unsuitable for transplantation can be used for research and surgical training purposes. The study was conducted according to the guidelines of the Declaration of Helsinki. Human eye bank corneas were preserved in corneal Iscove’s Modified Dulbecco’s Medium, and upon receipt they were immediately processed or kept at 4 °C for a maximum of 24 h until processing. All steps were conducted under a biological hood and sterile conditions. Briefly, after gently removing Descemet’s membrane and epithelium from the corneal button, the remaining stroma was minced in a Petri dish and digested overnight with 0.5 mg/mL collagenase (catalog no. C0130; Merck, Darmstadt, Germany) at 37 °C in a humidified CO_2_ incubator set to 5% CO_2_. Isolated cells were cultured in human corneal keratocyte growth medium (HCK Growth Medium Kit; catalog no. 6111K-500; Cell Applications, San Diego, CA, USA) supplemented with 15% fetal bovine serum (FBS), 100 U/mL penicillin, and 100 μg/mL streptomycin (catalog no. 15140122; Thermo Fisher Scientific, Waltham, MA, USA). After reaching confluency, cells were harvested and plated in 75 cm^2^ tissue flasks. Culture medium was changed every three days and cells were used between the third and fifth passages in culture for the different experiments.

### 4.2. Cell Stimulation

For stimulation experiments, cells were grown to 70% confluence, washed three times with serum-free medium and subsequently serum-starved overnight in human corneal keratocyte basal medium (HCK Basal Medium; catalog no. 6110-500; Cell Applications) supplemented with 2% FBS. The following day, 2 h after the addition of the sGC stimulator BAY 41-2272 (catalog no. B8810; Sigma-Aldrich, St. Louis, MO, USA) at a final concentration of 10 µM, cells were stimulated with recombinant human TGFβ1 (10 ng/mL; PeproTech, Rocky Hill, NJ, USA). BAY 41-2272 was dissolved in dimethyl sulfoxide (DMSO). The final concentration of DMSO in the experiments did not exceed 0.1%. Forty-eight h after TGFβ1 stimulation, cells were assayed for cell viability and proliferation, Matrigel chemoinvasion and gene expression, while 72 h after stimulation cells were analyzed for protein expression and contractile ability.

### 4.3. Annexin V/PI Flow Cytometry Assay

Keratocytes were grown up to 70% confluence, serum-starved overnight and then challenged as described above for 48 h. After stimulation, 1 × 10^5^ cells/tube were harvested with Accutase (Euroclone, Milan, Italy), incubated 15 min at 4 °C in the dark with 100 mL annexin binding buffer (100 mM 4-(2-hydroxyethyl)-1-piperazineethanesulfonic acid), 140 mM sodium chloride, 25 mM calcium chloride, pH 7.4), 1 mL of 100 mg/mL PI (Sigma-Aldrich) working solution and 5 mL annexin V allophycocyanin (APC)-conjugated (ImmunoTools, Friesoythe, Germany). Samples were analyzed with a BD FACS Canto II flow cytometer (BD Biosciences, Franklin Lakes, NJ, USA). Cellular distribution depending on annexin V and/or PI positivity allowed the measurement of the percentage of viable cells (annexin V^−^ and PI^−^), early apoptosis (annexin V^+^ and PI^−^), late apoptosis (annexin V^+^ and PI^+^), and necrosis (annexin V^−^ and PI^+^). The assay was performed in triplicate for each of the five cell lines. A minimum of 10,000 events were collected.

### 4.4. Cell Proliferation Assay

Isolated keratocytes were seeded onto 96-well plates (10 × 10^3^ cells per well) in HCK complete medium and left to adhere overnight. Cells were then washed three times with serum-free medium, incubated in 2% FBS-HCK basal medium for additional 24 h and finally stimulated for 48 h in 2% FBS-HCK basal medium supplemented with BAY 41-2272 alone, TGFβ1 alone, or TGFβ1 added 2 h after preincubation with BAY 41-2272. The proliferative effect with 2% FBS-HCK basal medium was set as basal growth. Cell proliferation was determined by WST-1 (4-[3-(4-iodophenyl)-2-(4-nitrophenyl)-2H-5-tetrazolio]-1,3-benzene disulfonate) assay (Merck) according to the manufacturer’s instructions. All measurements were performed in triplicate for each of the five cell lines, and the results were expressed as the percentage of the increase/decrease in cell proliferation over the basal response.

### 4.5. In Vitro Wound Healing Assay

Keratocytes were seeded onto 6-well tissue culture plates and cultured in HCK complete medium. Once they reached 80–90% confluence, cells were rinsed three times with serum-free medium and serum-starved overnight. The day after, the medium was removed, and the monolayer was scratched with a sterile 200-μL pipette tip in order to create a ~1 mm wide wound. After removing detached cells by carefully washing with HCK basal medium, the monolayer was incubated for 24 h with HCK 2% FBS-HCK basal medium supplemented with BAY 41-2272 alone, TGFβ1 alone, or TGFβ1 added 2 h after preincubation with BAY 41-2272. No mitosis inhibitors, such as mitomycin C, were employed in the assay. The healing capacity was assessed by capturing phase-contrast images of the wounded area at the beginning and after 24 h under a Leica inverted microscope (Leica Microsystems, Mannheim, Germany) with a ×10 objective. For each experimental point, images at 0 and 24 h were compared to quantify the cellular migration rate after wounding. All experimental conditions were performed in triplicate for each of the five cell lines.

### 4.6. Matrigel Chemoinvasion Assay

Chemoinvasion was assessed by using the Boyden chamber assay, which was performed in 24-well plates with inserts containing an 8 μm pore size polyethylene terephthalate membrane filter coated with Matrigel polymeric extracellular matrix (Corning BioCoat Growth Factor Reduced Matrigel Invasion Chamber; catalog no. 354483; Corning, Glendale, AZ, USA) separating the upper and lower wells of the migration chamber. TGFβ1 was placed in the lower wells as chemoattractant, while 25 × 10^3^ cells previously stimulated with TGFβ1, TGFβ1 and BAY 41-2272, or BAY 41-2272 alone for 48 h were suspended in 2% FBS-HCK basal medium and added in the upper wells. A chemokinetic effect was excluded by using 2% FBS-HCK basal medium in both the upper and the lower well (i.e., under this condition we failed to detect any cells on the lower side of the membrane). No mitosis inhibitors were employed in the assay. All experimental conditions were performed in triplicate for each of the five cell lines. Twenty-four hours after cell seeding, non-migrated cells were mechanically removed from the upper surface of the membranes with a cotton-tipped swab, while migrated cells, adherent on the lower filter surfaces, were fixed and subsequently stained with a Diff-Quik stain kit (Dade Behring, Deerfield, IL, USA) following the manufacturer’s instructions. The membranes were then washed with phosphate buffered saline (PBS), detached from the insert by means of a blade and finally mounted upside-down on glass slides. Each membrane was photographed under a Leica DM4000 B microscope (Leica Microsystems) equipped with a Leica DFC310 FX 1.4-megapixel digital color camera furnished with the Leica software application suite LAS V3.8 (Leica Microsystems). Migrated cells were counted in a blind manner by two independent observers.

### 4.7. RNA Purification, cDNA Synthesis and Quantitative Real-Time PCR

Forty-eight hours after TGFβ1 stimulation, cells were harvested and total RNA was isolated using the RNeasy Micro Kit (Qiagen, Milan, Italy). First, strand cDNA was synthesized using the QuantiTect Reverse Transcription kit (Qiagen), while mRNA quantification by SYBR Green real-time PCR was performed using the StepOnePlus Real-Time PCR System (Applied Biosystems, Milan, Italy), as described elsewhere [24,44]. Predesigned oligonucleotide primer pairs were obtained from Qiagen (QuantiTect Primer Assay). The assay IDs were Hs_ACTA2_1_SG (catalog no. QT00088102), Hs_COL1A1_1_SG (catalog no. QT00037793), Hs_COL1A2_1_SG (catalog no. QT00072058), Hs_FN1_1_SG (catalog no. QT00038024), Hs_PDPN_1_SG (catalog no. QT01015084), Hs_IL1B_1_SG (catalog no. QT00021385), Hs_IL6_1_SG (catalog no. QT00083720), Hs_TNF_1_SG (catalog no. QT00029162), and Hs_RRN18S_1_SG (catalog no. QT00199367). The PCR mixture was composed by 1 μL cDNA, 0.5 μM sense and antisense primers, 10 μL 2× QuantiTect SYBR Green PCR Master Mix containing SYBR Green I dye, ROX passive reference dye, HotStarTaq DNA Polymerase, dNTP mix and MgCl_2_ (Qiagen). Amplification was performed according to a standard protocol recommended by the manufacturer. Non-specific signals produced by primer dimers or genomic DNA were excluded by dissociation curve analysis, non-template controls, and samples without enzyme in the reverse transcription step. In all samples, 18S ribosomal RNA was measured as an endogenous control to normalize for the amounts of loaded cDNA. Differences were calculated with the threshold cycle (Ct) and comparative Ct method for relative quantification. All measurements were performed in triplicate for each of the five cell lines.

### 4.8. Western Blotting

Keratocyte protein lysates were obtained after 72 h upon TGFβ1 stimulation. Whole cell protein lysates were prepared as described elsewhere [24,44], and the amount of proteins in the samples was determined by the Micro BCA Protein Assay Kit (catalog no. 23235; Thermo Fisher Scientific, Waltham, MA, USA). After the addition of Laemmli sample buffer (Bio-Rad, Hercules, CA, USA) and β-mercaptoethanol, 40 µg of total proteins were boiled at 100 °C for 5 min, electrophoresed on precast polyacrylamide gels (4-15% Mini-Protean TGX Gels; Bio-Rad) and blotted onto nitrocellulose membranes (Bio-Rad). The membranes were blocked for 30 min at room temperature in 5% *w*/*v* milk buffer (5% *w*/*v* non-fat dried milk, 50 mM Tris, 200 mM NaCl, 0.2% Tween-20) and subsequently incubated overnight at 4 °C with the following primary antibodies diluted in 5% *w*/*v* milk buffer: mouse monoclonal anti-α-SMA (1:300 dilution; catalog no. ab7817; Abcam, Cambridge, UK), rabbit monoclonal anti-COL1A1 (1:1000 dilution; catalog no. #39952; Cell Signaling Technology, Danvers, MA, USA), rabbit monoclonal anti-N-cadherin (1:1000 dilution; catalog no. #13116S; Cell Signaling Technology), mouse monoclonal anti-vimentin (1:1000 dilution; catalog no. M7020; Dako, Glostrup, Denmark), mouse monoclonal anti-PDPN (1:200 dilution; catalog no. ab10288; Abcam), rabbit polyclonal anti-Smad3 (1:1000 dilution; catalog no. #9513S; Cell Signaling Technology), rabbit monoclonal anti-p-Smad3 (Ser423/425) (1:1000 dilution; catalog no. #9520S; Cell Signaling Technology), rabbit polyclonal anti-α-tubulin (1:1000 dilution; catalog no. #2144; Cell Signaling Technology), rabbit polyclonal anti-α-actinin (1:1000 dilution; catalog no. #3134; Cell Signaling Technology), and mouse monoclonal anti-glyceraldehyde 3-phosphate dehydrogenase (GAPDH) (1:5000 dilution; catalog no. ab8245; Abcam). α-tubulin, α-actinin, and GAPDH were assumed as control invariant proteins. The immunoblots were washed three times in Tris buffered saline (Bio-Rad) with 0.1% Tween-20, and then incubated for 1 h at room temperature with HRP-conjugated secondary antibodies (Cell Signaling Technology). The proteins were visualized by an enhanced chemiluminescence method (Clarity Western ECL Substrate; Bio-Rad) and analyzed by ChemiDoc Touch Imaging System (Bio-Rad). Band intensities were quantified using the free-share ImageJ software (NIH, Bethesda, MD, USA; online at http://rsbweb.nih.gov/ij, accessed on 16 May 2022) and values were normalized to α-tubulin, α-actinin or GAPDH, as needed. The assay was performed in duplicate for each of the five cell lines.

### 4.9. Immunofluorescence

Keratocytes, seeded onto glass coverslips and treated as previously described for 72 h, were fixed with 3.7% buffered paraformaldehyde, permeabilized with 0.1% Triton X-100 in PBS and washed with PBS. Slides were then blocked with 1% bovine serum albumin in PBS for 1 h at room temperature, and finally incubated overnight at 4 °C with the following primary antibodies: anti-CD34 (1:50 dilution; catalog no. ab81289; Abcam), anti-α-SMA (1:100 dilution; catalog no. ab7817; Abcam), anti-COL1A1 (1:300 dilution; catalog no. #39952; Cell Signaling Technology), or anti-podoplanin (1:200 dilution; catalog no. ab10288; Abcam). The day after, slides were incubated for 45 min at room temperature in the dark with Alexa Fluor-488-conjugated or Rhodamine Red-X-conjugated secondary antibodies (1:200 dilution; Invitrogen). Irrelevant isotype-matched and concentration-matched mouse and rabbit IgG (Sigma-Aldrich) were used as negative controls. Nuclei were counterstained with 4′,6-diamidino-2-phenylindole (DAPI). Immunostained cells were examined with a Leica DM4000 B microscope (Leica Microsystems) and fluorescence images were captured with a Leica DFC310 FX 1.4-megapixel digital color camera equipped with the Leica software application suite LAS V3.8 (Leica Microsystems). The assay was performed in duplicate for each of the five cell lines.

### 4.10. Collagen Gel Contraction Assay

Collagen gel contraction assay was performed using the CytoSelect 24-Well Cell Contraction Assay Kit (Floating Matrix Model; catalog no. CBA-5020; Cell Biolabs, San Diego, CA, USA) according to the manufacturer’s instructions. Keratocytes were harvested, pelleted and resuspended in serum-free medium at 5 × 10^6^ cells/mL. Cells were treated as previously described for 48 h before the assay. For each experimental point, 100 µL of cell suspension were mixed with 400 µL of cold neutralized collagen gel solution, and subsequently added to one well of the adhesion resistant matrix-coated 24-well cell contraction plate. Gels were allowed to solidify for 1 h at 37 °C in a humidified CO_2_ incubator set to 5% CO_2_. After polymerization, 1 mL of basal media or media containing different stimuli was added to the top of each collagen gel lattice. Each experimental point was performed in triplicate for the five cell lines. After 24 h, the culture dish was scanned, and the area of each collagen gel was measured by ImageJ software (NIH).

### 4.11. Statistical Analysis

Statistical analysis was performed using the Statistical Package for Social Sciences (SPSS) software for Windows, version 28.0 (SPSS, Chicago, IL, USA). A total of 15 data points (i.e., 5 cell lines assessed in 3 replicates) were included into the statistical analysis for all the assays, except for Western blotting for which a total of 10 data points (i.e., 5 cell lines assessed in 2 replicates) were analyzed. Data are expressed as the mean ± standard error of the mean (SEM). After assessing the normality of data by Kolmogorov–Smirnov test, a one-way ANOVA with post-hoc Tukey’s test was used for statistical analyses. Values of *p* < 0.05 were considered statistically significant.

## Data Availability

All relevant data are included within the manuscript.
